# Carvacrol and *Zataria multiflora* influenced the PPARγ agonist effects on systemic inflammation and oxidative stress induced by inhaled paraquat in rat 

**DOI:** 10.22038/ijbms.2020.45962.10648

**Published:** 2020-07

**Authors:** Fatemeh Amin, Arghavan Memarzia, Hamid Reza Kazerani, Mohammad Hossein Boskabady

**Affiliations:** 1 Department of Physiology, Faculty of Veterinary Medicine, Ferdowsi University of Mashhad, Mashhad, Iran.; 2 Department of Physiology, Faculty of Medicine, Mashhad University of Medical Sciences, Mashhad, Iran; 3 Neurogenic Inflammation Research Center, Mashhad University of Medical Sciences, Mashhad, Iran

**Keywords:** Carvacrol, Oxidative stress, Paraquat, PPAR-γ agonist, Systemic inflammation, Zataria multiflora

## Abstract

**Objective(s):**

The effects of PPAR-γ agonist alone and in combination with carvacrol and *Zataria* *multiflora* on inhaled paraquat (PQ) induced-systemic inflammation and oxidative stress were examined.

**Materials and Methods:**

Control group exposed to normal saline aerosol, one group exposed to 54 mg/m^3^ PQ aerosol and four groups exposed to PQ aerosol and treated with 5 mg/kg/day pioglitazone, pioglitazone + 200 mg/kg/day Z. *multiflora* extract, pioglitazone + 20 mg/kg/day carvacrol, and 0.03 mg /kg/day dexamethasone for 16 days after the end of exposure to PQ were studied. Exposure to normal saline or PQ was performed every other days for 30 min (8 times). Different variables were measured after the end of treatment period.

**Results:**

PQ exposure significantly increased serum levels of NO_2_, MDA and IL-6 but dexreased CAT and IFN-γ levels and IFN-γ/IL-6 ratio compared to control group (*P*<0.01 to *P*<0.001). Treatment with pioglitazone only improved serum level of MDA (*P*<0.01). Treatment with combination of pioglitazone and carvacrol as well as treatment with dexamethasone improved all measured variables compared to PQ exposed group (*P*<0.05 to *P*<0.001). The effects of pioglitazone + Z. *multiflura* and pioglitazone + carvacrol on almost all measured variables were significantly higher than pioglitazone alone (*P*<0.05 to *P*<0.001).

**Conclusion:**

The effects of combination therapy of pioglitazone with Z. *multiflora* or carvacrol on inhaled paraquat (PQ) induced-oxidative stress and systemic inflammation were higher than the effects of pioglitazone alone. These results suggested that the effects of the extract and carvacrol may mediated through PPAR-γ receptors.

## Introduction

Although agrochemicals such as herbicides, fertilizers and pesticides are an important factor in increasing the products yield in agriculture, negative effects of these compounds on human health is undeniable (1). Paraquat (PQ) is a nonselective herbicide which inducing inflammation and oxidative stress in various organs (2). This herbicide is highly used for control of more than 100 different crops including corn, rice, soybeans, vegetables, orchards, and many other crops in about 100 countries in the global agricultural field, especially in Asian countries (3). 

Paraquat is used as herbicide in agriculture in nearly 130 countries, especially in third world countries and the rate of death from pesticide poisoning is generally, 250,000 to 370,000 each year. The disability-adjusted life years (DALY) in the northeast of Colombia by PQ poisoning was around 53.4 years per 100,000 inhabitants in 2019 but in 2015 it was higher (26,900 DALYs per 100,000), (4). In Iran, the incidence of acute PQ poisoning from 2004 to 2013 was 30,485 (3049 per year) which was increased from 1808 in 2004 to 4283 in 2013 and years of life lost (YLL) was 20,709 in this period of time (5).

It was shown that PQ is highly toxic for human and most animals (6). Small dose of PQ (30 mg/kg) is lethal in adult human (7) due to generation of intracellular reactive oxygen species, redox reactions and lipid peroxidation of cellular membranes (8). Systemic inflammation induced by inhaled PQ is related to actively absorbance of its particles which causes leukocytosis, pulmonary hypertension, heart enlargement, acute renal damage, edema and increasing amylase, glucose and creatinine (9). It has also been shown that exposure to PQ can lead to an increase in inflammatory factors such as interleukins and TNF-α (9). PQ poisoning and its lethal effects makes it become a severe public health challenge. Therefore, preventing systemic absorption and the effect of PQ is one of the important issues in human health. The current treatment of acute PQ poisoning is the prescribing activated charcoal. Moreover, some anti-oxidants- such as acetylcysteine and salicylate, anti-inflammatory and immunosuppressive agents are also used as potential treatments for PQ poisoning (8). 

There is no effective therapeutic drugs has yet been introduced to prevent PQ-induced systemic toxicity (3, 10). However, the available treatment for PQ poisoning includes using immunosuppressive drugs such as dexamethasone, cyclophosphamide and methylprednisolone. Acetylcysteine and salicylate as anti-oxidant agents are also of therapeutic values by reducing free radical scavenging, anti-inflammatory and NF-kB inhibitory actions. However, the therapeutic efficacy of these drugs are very weak and despite the mentioned treatments, there is a high rate of mortality due to acute PQ poisoning (11).

Peroxisome proliferator-activated receptors (PPARs) are nuclear, ligand-activated transcription factors receptor that have effective role in improvement of various diseases such as inflammatory disorders, cancer and lung diseases (12). Up to now, three PPAR subtypes have been identified including: PPARα, PPAR β/δ, and PPARγ (13). PPARγ receptor is the main target of thiazolidinediones (TZDs). This drugs are frequently used to combat the hyperglycemia state which can be associated with metabolic syndrome (14). It was shown that some natural or synthetic PPARγ ligands such as prostaglandins, eicosanoids, fatty acids, leukotrienes, rosiglitazone, and pioglitazone (15) are capable of regulating cellular metabolism, differentiation, apoptosis and inflammation via modulating of different gene products (14, 16).

*Zataria multiflora* Boiss (*Z. multiflora*) belongs to the Lamiaceae family. This medicinal herb grows only in the south and the central regions of Iran, Afghanistan and Pakistan (17). Traditionally, this plant have been used as a remedy against the lung inflammation (18). *Z. multiflora* extract contains carvacrol and thymol, two active ingredients with strong anti-oxidant properties that have the potential to be used in inflammatory and immune deficiency diseases associated with increased oxidative stress (18). *Z. multiflora* and its constituent carvacrol also showed anti-oxidant and anti-inflamatory effects by scavenging free radicals, as well as the protective effects on malondialdehyde (19). 

Although the effects of PPARγ agonists, *Z. multiflora* extract and carvacrol on the treatment of inflammatory and immune-dysregulation disorders were reported, no available study has evaluated the effects of these compounds on systemic toxicity induced by PQ inhalation. Hence the aims of the present study are exploring the potential effects of PPARγ agonist alone and in combination with *Z. multiflora* hydroalcoholic extract and carvacrol on the PQ inhalation induced systemic toxicity in the male Wistar rat.

## Materials and Methods

***Animal and experimental groups***

Thirty male Wistar rats (weighing 200–250 g) were purchased and keep in the animal house, School of Medicine, Mashhad University of Medical Sciences, Iran in standard condition (22±2 ^°^C temperature, 12 hr light/dark cycle and diet and tap drinking water *ad libitum*). In the present study only male rats with weight range of 200-250 g were studied as inclusion criteria, and female rats with low or high weight were considered as exclusion criteria. Experiments were done according to criteria outlined in the Guide for Care and Use of Laboratory Animals (NIH US publication 23-68 revised 1985; ttp://oacuod.nih.gov/regs/guide/guidex.htm). The study was approved by the Ethics Committee of Mashhad University of Medical Sciences for Animal Experiments (code 961202).

Rats were randomly assigned into six groups (n=5 in each group) as: 1) Exposed rats to aerosol of normal saline, every other day for 16 days, 8 times, (control group). 2) Exposed rats to aerosol of PQ (Sigma Aldrich Co, China), (20). 3) Exposed rats to aerosol of PQ and treated with 5 mg/kg/day pioglitazone (Samisaz Pharmaceutical Company, Iran), by gavage for 16 days after the end of PQ exposure period (21). 4) Exposed rats to aerosol of PQ and treated with pioglitazone + 200 mg/kg/day *Z. multiflora* hydroalcoholic extract by gavage for 16 days after the end of PQ exposure period. 5) Exposed rats to aerosol of PQ and treated with pioglitazone + 20 mg/kg/day carvacrol by gavage for 16 days after the end of PQ exposure period (22). 6) Exposed rats to aerosol of PQ and treated with 0.03 mg/kg/day dexamethasone (Sigma, St. Louis, MO, Germany), by gavage for 16 days after the end of PQ exposure period (23). Carvacrol and pioglitazone were diluted in saline by adding few drop of Tween-80 to make their appropriate concentrations for gavage. In control and PQ exposed groups also the same volume of saline (5 ml) as the extract, carvacrol and pioglitazone was gavaged in the same manner as treated groups. 

***Exposure to paraquat***

**Figure 1:**
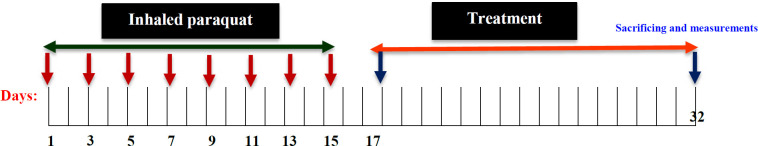
Protocol of exposing animals to inhaled PQ and treatment with pioglitazone or combination of pioglitazone with *Z. multiflora* or carvacrol

PQ aerosol was delivered to animal head box dimensions 15×18×30 cm for eight times in one other day order, totally for 16 days (for 30 min/day). PQ aerosol was produced by a nebulizer (Omron CX3, Japan, particle size 3-5 μm) with an air flow of 8 lit/min. Each time, ‌4.5 ml of 1.33 mg/ml PQ (Sigma Aldrich Co, China) solution was added to the nebulizer chamber. The solution volume output of the nebulizer was 0.15 ml/min and its air output was 3.7 l/min. Therefore the PQ dose in the aerosol was 54 mg/m^3^ (20, 23). Pioglitazone alone or in combination with *Z. multiflora* extract or carvacrol as well as dexamethasone were daily gavaged after the end of PQ exposure for16 days. In control group, saline was used instead of the PQ solution (24), (Figure 1).

***Plant and extract***

The plant was collected (Herbarium No: 35314, FUMH) and the aqueous-ethanolic extract of the plant was prepared as described previously (25). Dried extract was added to water in order to prepare daily fresh extract for gavage. In our previous study, the characteristic of the extract of *Z. multiflora* was identified (26). 

***Oxidant, and anti-oxidant biomarkers measurement***

Serum levels of oxidants including MDA and NO_2_ were measured as described in a previous study (27). 

The activity of CAT enzyme in serum as an anti-oxidant marker was measured based on the method described previously (27).

***Cytokines measurement***

Serum levels of IL-6 and IFN-γ were measured using specific enzyme-linked immunosorbent assay (ELISA) kits (Hangzhou Eastbiopharm, Iran) based on the manufacture technique as previously described (28). 

***Statistical analysis***

The results were presented as mean±SEM and were analyzed using one-way analysis of variance (ANOVA) followed by Tukey’s multiple comparison test. Statistically significance were considered when *P*-value was less than 0.05 or lower.

## Results

***The effects of paraquat exposure***

**Figure 2:**
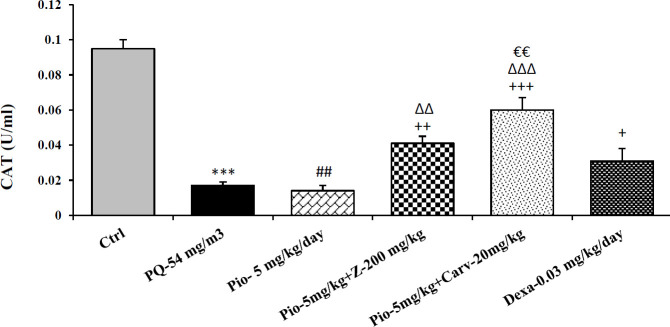
Serum level of catalase (CAT) in different studied groups. The results are expressed as mean±SEM (n=5 in each group). ***P<0.01 compared to the control group. ++*P*<0.01 and +++*P*<0.001compared to the PQ group, ##*P*<0.01 compared to dexamethasone treated group. ∆∆*P*<0.01 and ∆∆∆*P*<0.001 compared to pioglitazone treated group. €€P<0.01 compared to group treated with combination of pioglitazone ant the extract. Comparisons between different groups were made using one-way ANOVA followed by Tukey’s multiple comparison test. Ctrl, control group, PQ-54 mg/m3, group exposed to paraquat aerosol with dose of 54 mg/m^3^, Pio-5 mg/kg/day, Pio-5 + Z- 200 mg/kg/day, Pio-5 + Carv-20 mg/kg/day and Dex-0.03mg/kg/day, groups exposed to PQ-54 mg/m^3^ and treated with 5 mg/kg/day pioglitazone, combination or 200 *Zataria multiflora* and 20 mg/kg/day and carvacrol respectively or dexamethasone respectively

**Figure 3.**
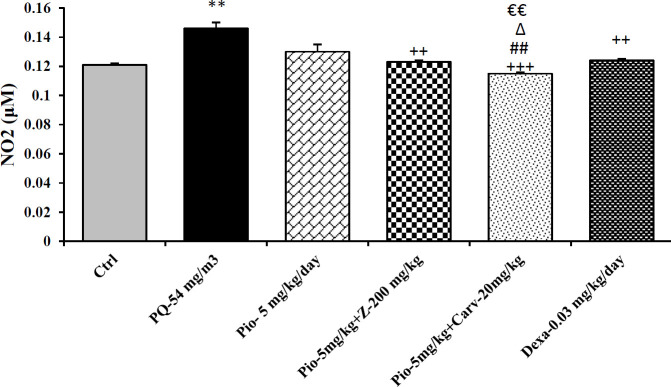
Serum level of nitrite (NO2) in different studied groups. The results are expressed as mean±SEM (n=5 in each group). **P<0.01 compared to the control group. ++*P*<0.01 and +++*P*<0.001 compared to the PQ group. ##*P*<0.01 compared to dexamethasone treated group. ∆*P*<0.05 compared to pioglitazone treated group. €€*P*<0.01 compared to group treated with combination of pioglitazone ant the extract. Comparisons between different groups were made using one-way ANOVA followed by Tukey’s multiple comparison test. Ctrl, control group, PQ-54 mg/m3, group exposed to paraquat aerosol with dose of 54 mg/m^3^, Pio-5 mg/kg/day, Pio-5 + Z- 200 mg/kg/day, Pio-5 + Carv-20 mg/kg/day and Dex-0.03mg/kg/day, groups exposed to PQ-54 mg/m^3^ and treated with 5 mg/kg/day pioglitazone, combination or 200 *Zataria multiflora* and 20 mg/kg/day and carvacrol respectively or dexamethasone respectively

**Figure 4:**
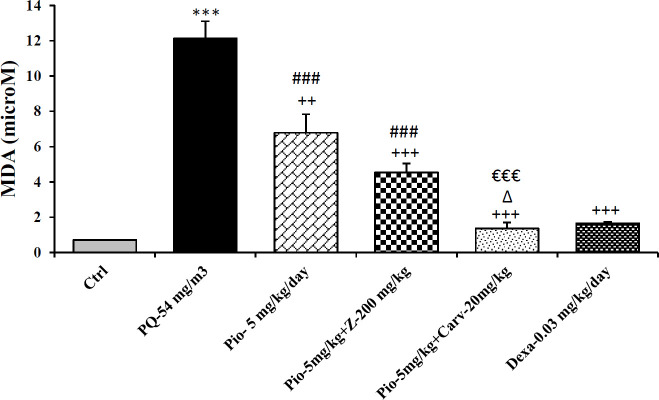
Serum level of malondialdehyde (MDA) in different studied groups. The results are expressed as mean±SEM (n=5 in each group). ***P<0.001 compared to the control group. ++ *P*<0.01 and +++*P*<0.001 compared to the PQ group. ###*P*<0.001 compared to dexamethasone treated group. ∆*P*<0.05 compared to pioglitazone treated group. €€€*P*<0.001 compared to group treated with combination of pioglitazone and the extract. Comparisons between different groups were made using one-way ANOVA followed by Tukey’s multiple comparison test. Ctrl, control group, PQ-54 mg/m^3^, group exposed to paraquat aerosol with dose of 54 mg/m^3^, Pio-5 mg/kg/day, Pio-5 + Z- 200 mg/kg/day, Pio-5 + Carv-20 mg/kg/day and Dex-0.03mg/kg/day, groups exposed to PQ-54 mg/m^3^ and treated with 5 mg/kg/day pioglitazone, combination or 200 *Zataria multiflora* and 20 mg/kg/day and carvacrol respectively or dexamethasone respectively

**Figure 5:**
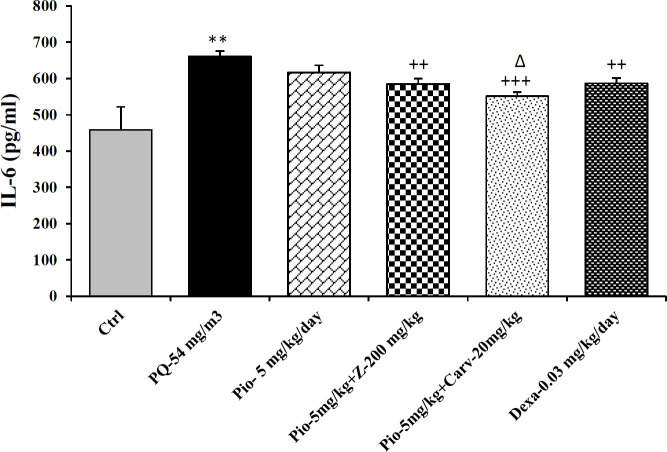
Serum level of interlocking-6 (IL-6) in different studied groups. The results are expressed as mean±SEM (n=5 in each group). **P<0.01 compared to the control group. ++*P*<0.01 and +++*P*<0.001 compared to the PQ group. ∆*P*<0.05 compared to pioglitazone treated group. Comparisons between different groups were made using one-way ANOVA followed by Tukey’s multiple comparison test. Ctrl, control group, PQ-54 mg/m^3^, group exposed to paraquat aerosol with dose of 54 mg/m^3^, pio-5 mg/kg/day, Pio-5 mg/kg/day, Pio-5 + Z- 200 mg/kg/day, Pio-5 + Carv-20 mg/kg/day and Dex-0.03mg/kg/day, groups exposed to PQ-54 mg/m^3^ and treated with 5 mg/kg/day pioglitazone, combination or 200 *Zataria multiflora* and 20 mg/kg/day and carvacrol respectively or dexamethasone respectively

**Figure 6:**
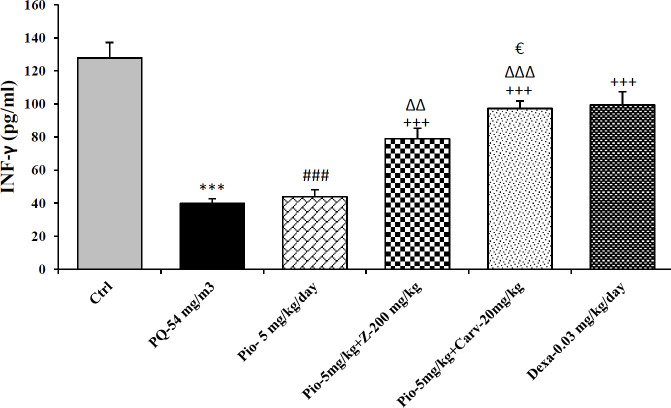
Serum level of Interferon gama (INF-γ) in different studied groups. The results are expressed as mean±SEM (n=5 in each group). ***P<0.001 compared to the control group. +++*P*<0.001 compared to the PQ group. ###*P*<0.001 compared to dexamethasone treated group. ∆∆P<0.01 and ∆∆∆*P*<0.001compared to pioglitazone treated group. €*P*<0.05 compared to treated group with combination of pioglitazone ant the extract. Comparisons between different groups were made using one-way ANOVA followed by Tukey’s multiple comparison test. Ctrl, control group, PQ-54 mg/m^3^, group exposed to paraquat aerosol with dose of 54 mg/m^3^, Pio-5 mg/kg/day, Pio-5 mg/kg/day, Pio-5 + Z- 200 mg/kg/day, Pio-5 + Carv-20 mg/kg/day and Dex-0.03mg/kg/day, groups exposed to PQ-54 mg/m^3^ and treated with 5 mg/kg/day pioglitazone, combination or 200 *Zataria multiflora* and 20 mg/kg/day and carvacrol respectively or dexamethasone respectively

**Figure 7:**
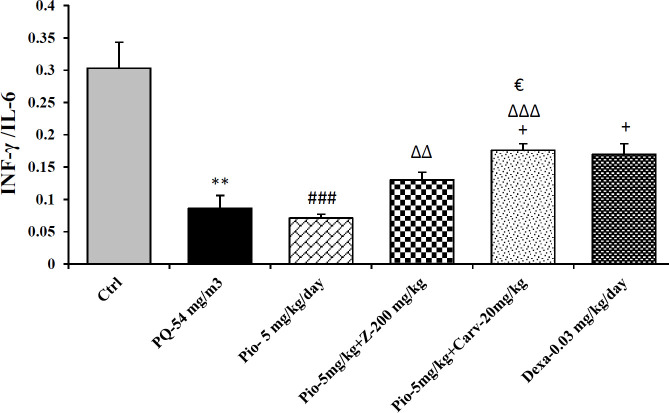
IFN-γ/IL-4 ratio in different studied groups. The results are expressed as mean±SEM (n=5 in each group). **P<0.01 compared to the control group. +*P*<0.05 compared to the PQ group. ###*P*<0.001 compared to dexamethasone treated group. ∆∆ *P*<0.01 and ∆∆∆ *P*<0.001 compared to pioglitazone treated group. € *P*<0.05 compared to treated group with combination of pioglitazone ant the extract. Comparisons between different groups were made using one-way ANOVA followed by Tukey’s multiple comparison test. Ctrl, control group, PQ-54 mg/m^3^, group exposed to paraquat aerosol with dose of 54 mg/m3, Pio-5 mg/kg/day, Pio-5 + Z- 200 mg/kg/day, Pio-5 + Carv-20 mg/kg/day and Dex-0.03 mg/kg/day, groups exposed to PQ-54 mg/m^3^ and treated with 5 mg/kg/day pioglitazone, combination or 200 *Zataria multiflora* and 20 mg/kg/day and carvacrol respectively or dexamethasone respectively

Inhaled PQ (54 mg/m^3^) caused significant increase in NO_2_ and MDA serum levels but decrease CAT activity compare to control group (*P*<0.01 for NO_2_, *P*<0.001 for MDA and CAT). Exposure of rats to inhaled PQ (54 mg/m^3^) caused significant increased IL-6 level but decreased INF-γ level and IFN-γ/IL-6 ratio compared to control group (*P*<0.01 for IL-6 and IFN-γ/IL-6 ratio but *P*<0.001 for IFN-γ), (Figures 2-7).

***The effects of pioglitazone***

Treatment with pioglitazone (5 mg/kg) significantly decrease serum level of MDA compared to PQ group (*P*<0.01). However, other measured parameters did not significantly improved due to pioglitazone treatment compared to PQ group (Figures 2-7).

***The effects of combination treatment with pioglitazone and the extract or carvacrol***

The combination therapy of pioglitazone and *Z. multiflura* extract improved serum levels of CAT, NO_2_, MDA and IL-6 compared to PQ exposed group (*P*<0.001 for MDA and *P*<0.01 for other cases), (Figures 2-7). However, treatment with combination of pioglitazone and carvacrol improved all measured variables compared to PQ exposed group (*P*<0.05 for IFN-γ/IL-6 ratio and *P*<0.001 for other cases), (Figures 2-7).

The effects of combination therapy of pioglitazone and *Z. multiflura* extract on serum levels of CAT and INF-γ as well as IFN-γ/IL-6 ratio were higher than treatment effect of pioglitazone alone (*P*<0.01 for all cases), (Figures 2, 6 and 7). All measured variables in the treatment group with combination of pioglitazone and carvacrol were significantly higher than pioglitazone alone treated group *P*<0.05 for NO_2_, MDA, IL-6 and *P*<0.001 for other cases), (Figures 2-7). In addition, the effects of treatment with combination of pioglitazone and carvacrol on serum levels of all measured variables except IL-6 were higher than the treatment effects of combination with pioglitazone and *Z.multiflura *(*P*<0.05 to *P*<0.001), (Figures 2-3, 5 and 6).

***The effects of dexamethasone***

Dexamethasone treatment caused significant improvement in all measured variables compared to PQ exposed group (*P*<0.05 for CAT and IFN-γ/IL-6 ratio, and *P*<0.01 for NO_2_ and IL-6 and *P*<0.001 for other cases), (Figures 2-7). The effects of dexamethasone treatment on serum levels of CAT, MDA IFN-γ and IFN-γ/IL-6 ratio were higher than the effects of treatment with pioglitazone (*P*<0.01 for CAT and *P*<0.001 for other cases), (Figures 1, 3, 4, 6 and 7). The effect of treatment with dexamethasone on serum level of MDA was also higher than the effect of combination therapy of pioglitazone and *Z. multiflora extract *(*P*<0.001), (Figure 4). However, the effect of dexamethasone therapy was lower than the effect of combination therapy of pioglitazone and carvacrol on serum level of NO_2_ (*P*<0.001), (Figure 3).

## Discussion

The results indicated that exposure to aerosols of PQ (54 mg/m^3^) in rat, increased serum level of NO_2_ and MDA, but serum CAT activity was decreased. Serum level of IL-6 was also increased but IFN-γ level in the serum, and IFN-γ/IL-6 ratio were decreased in rats exposed to inhaled PQ.

Various experiments and clinical studies have shown, that free radicals generation play a crucial role of PQ-induced injuries and it was shown that the major mechanism of acute and chronic toxicity by paraquat occurred through oxidative stress (29). In addition, significant rise in lipid peroxidation level in the lung tissues was observed with PQ administration in the animals which was accompanied by decreases in SOD and CAT activities (30). Decreased levels of SOD, CAT and increased levels of MDA and NO_2_ were showed due to PQ toxicity (31), Positive correlation was also observed between the enhanced levels of oxidants and inflammatory mediators with PQ administration doses (32) which support the findings of the present study regarding the induction of oxidative stress induced by inhaled PQ.

It was also shown that increased inflammatory cytokines play an important role in paraquat poisoning (33). Increased white blood cell (WBC), neutrophil count (3), and decreased anti-inflammatory cytokines such as IL-10 and IFN-γ, demonstrate inflammatory process caused by paraquat toxicity (2). In addition previous studies demonstrated enhanced genes expression IL-4, TGF-β, IL-17, (34) and TNF-α after PQ challenge (35), which support the results of this study indicating systemic inflammation induced by inhaled PQ.

The current therapy of paraquat poisoning are not effective, which indicated a major need for novel therapies for disorders induced by PQ exposure. In the last decade, researchers have been focused on the use of anti-oxidant and anti-inflammatory compounds for treatment of PQ toxicity. Evidence showed that activation of PPAR-γ receptors reduces inflammation and oxidative stress in various tissues, including the kidney, nervous system, and liver (36), Also it has been observed that the PPAR-γ agonist pioglitazone improved anti-oxidant capacity, and increases SOD and CAT enzymes production in a kidney ischemia-reperfusion model (37). PPAR-γ agonists inhibit nitric oxid, TNF-α, IL-1β, IL-6 levels, and MCP-1 from microglia and astrocytes (38). Decreased IL-6 and IL-8 in endometrial stromal cells via a PPAR-γ-dependent mechanism by thiazolidinediones was also reported. Therefore there is a positive association between activation of this receptors and suppression of inflammatory factors (39). 

In the present study, treatment with pioglitazone (5 mg/kg), PPAR-γ agonist only decrease serum level of MDA compared to PQ group and did not improved the other measured parameters in PQ exposed rats. These results could be due to inadequate dose of pioglitazone used in the present study. The results of a previous study also showed minor effect of treatment with 200 mg/kg/day *Z.multiflura* extract in animals exposed to inhaled PQ (23). Treatment with 20 mg/kg/day carvacrol, also showed minior effect on animals exposed to inhaled PQ.

However, treatment with combination of pioglitazone and *Z. multiflura* extract improved serum levels of CAT, NO_2_, MDA and IL-6 and treatment with combination of pioglitazone and carvacrol improved all measured variables compared to PQ exposed group. In addition, the effects of combination therapy of pioglitazone and *Z.multiflura* extract as well as the effect of pioglitazone and carvacrol were significantly higher than pioglitazone alone treated group. Therefore combination therapy of PPAR-γ agonist with *Z.multiflura* and specially its constituent, carvacrol improved oxidative stress, cytokines and increased IFN-γ/IL-6 ratio or Th1/Th2 balance in animals exposed to inhaled PQ. These results may indicate a synergism effect of the *Z.multiflura* and its constituent, carvacrol with the effect pioglitazone. 

Anti-oxidant and anti-inflammatory properties of *Z. multiflora* extract and its constituent carvacrol in animal and human studies were reported (18). The extract and carvacrol showed anti-oxidat and anti-inflammatory effects in a guinea pig model of asthma and COPD (40). In clinical studies also, *Z. multiflora* extract and carvacrol reduced MDA level as an oxidant marker and increased anti-oxidant levels including thiol, SOD and CAT (41). The plant and its constituent also showed anti-inflammatory effect in animal model of asthma (22). as well as in clinical studies (26, 41, 42). The effect of extract of *Z. multiflora* and carvacrol on enhanced IL-4/IFN-γ ratio as an indicator of Th1/Th2 balance were also shown previously which support the finding of the current study (34, 43, 44).

Therefore, the results may suggest that the effects of the plant and carvacrol could be mediated by their effects on PPAR-γ receptors. However, further studies, examining PPAR-γ antagonists needed to confirm this suggestion. In addition, the effects of treatment with combination of pioglitazone and carvacrol on all measured variables except IL-6 were higher than the treatment effects of combination with pioglitazone and *Z.multiflura *which suggest that the effect of the plant is due to its constituent, carvacrol.

In fact, Hotta *et al*. showed suppressing effect of carvacrol on COX-2 and activation of PPAR (45) which support the findings of the present study regarding the effect of *Z.multiflura* and carvacrol on PPAR-γ receptors.

Farmers are usually exposing to PQ through inhalation. Therefore, the aims of the present study are exploring the potential effects of PPARγ agonist alone and in combination with *Z. multiflora* hydro alcoholic extract and carvacrol on the PQ inhalation induced systemic toxicity in the male Wistar rat by examining oxidant, anti-oxidant markers and cytokine levels in the serum. The results showed significant changes in serum levels of all oxidant, anti-oxidant and cytokine indicating the induction of systemic oxidative stress and inflammation by inhaled PQ which are novel findings. However, the induction of lung inflammation and oxidative stress by inhaled PQ was shown in our previous study (23). Our previous study showed the minimal effect of 200 mg/kg *Z. multiflura* extract and 20 mg/kg carvacrol but significant effects of their 800 and 200 mg/kg on rats exposed to paraquat respectively (23). In the present study the low dose of the extract and carvacrol was used to evaluate their synergism effect with pioglithasone.

The results of the present study also showed similar effect of dexamethasone a known anti-inflammatory drug with the effects of the combination of pioglitazone with the extract and carvacrol on oxidative stress and inflammation process induced by inhaled PQ which is a further evidence for the anti-inflammatory and anti-oxidant effect of the plant and its constituent carvacrol.

PQ: Paraquat

**Table 1:** Studied groups, their exposing to saline or paraquate and treatment protocol

Groups	Exposed to Agent and duration	Dose	Treatment with Agent and duration	Dose

Control	Salin aerosol for 30/day min, 8 times in every other day order over 16 days	-	-	-
PQ	Paraquat aerosol for 30/day min, 8 times in every other day order over 16 days	54 mg/m^3^	-	-
PQ-Pio	Paraquat aerosol (54 mg/m^3^) for 30 min, 8 times in every other day order over 16 days	54 mg/m^3^	Pioglitazone for 16 days after the end of PQ exposure	5 mg/kg/day
Pio+Z	Paraquat aerosol for 30 min/day, 8 times in every other day order over 16 days	54 mg/m^3^	Pio* + Z. multiflora* extract for 16 days after the end of PQ exposure	Pio + 200 mg/kg/day
Pio+Carv	Paraquat aerosol for 30 min/day, 8 times in every other day order over 16 days	54 mg/m^3^	Pio + Carvacrol for 16 days after the end of PQ exposure	Pio + 20 mg/kg/day
PQ-Dexa	Paraquat aerosol for 30 min/day, 8 times in every other day order over 16 days	54 mg/m^3^	Dexamethasone for 16 days after the end of PQ exposure	0.03 mg/kg/day

## Conclusion

Combination therapy of pioglitazone with *Z. multiflora* extract or carvacrol reduced NO_2_ and MDA but increased CAT levels as well as reduced IL-6 but increased IFN-γ and Th1/Th2 balance in rats exposure to PQ, similar is to of the effect of dexamethasone. However, pioglitazone alone in the present study and the extract and carvacrol in previous study at studied doses showed minimum effects on changes induced by PQ aerosol. These results suggest that the effects of *Z. multiflora* extract or carvacrol are mediated by their effect on PPAR-γ receptor.

## Financial Disclosure

This work was financially supported by a grant from Research Council of Mashhad University of Medical Sciences and Ferdowsi University of Mashad, Mashad, Iran (Code: 961202), Mashhad, Iran. The results described in this article are a part of PhD, thesis of Fatemeh Amin.

## Conflicts of Interest

The authors declare that there are no conflicts of interest.
